# Comprehensive Analysis of Free BDPE Content in Commercial Hyaluronic Acid Fillers: Implications for Safety Assessment and Regulatory Standards

**DOI:** 10.1111/jocd.70790

**Published:** 2026-03-10

**Authors:** Cheol Joo Kim, Mirou Lee, Hyeongtaek Park, Jaeyoung Jo, Hyolim Lee, Pius J. Seo, Seung Jun Shin, Chulho Shin

**Affiliations:** ^1^ R&D Department Across Co. Ltd. Chuncheon Republic of Korea; ^2^ Department of Medical Affairs Hugel Inc. Seoul Republic of Korea

**Keywords:** BDDE, BDPE, cross‐linked hyaluronate, cross‐linker, dermal filler, sodium hyaluronate

## Abstract

**Background:**

Although manufacturers of 1,4‐butanediol diglycidyl ether (BDDE)‐cross‐linked hyaluronic acid (HA) fillers assert effective removal of unreacted BDDE, the hydrolyzed derivative 3,3′‐(butane‐1,4‐diyl)bis(oxy)bis(propane‐1,2‐diol) (BDPE) is routinely monitored, despite possessing structural features associated with sensitization potential.

**Aims:**

To quantify free BDPE content across commercially available HA dermal fillers and assess potential safety implications.

**Materials/Methods:**

A validated liquid chromatography–tandem mass spectrometry method was developed to quantify BDPE levels in 38 commercial HA filler products from seven major manufacturers. *In silico* prediction models were used to evaluate the skin sensitization and irritation potential of BDPE.

**Results:**

BDDE levels were non‐detectable in all analyzed products. By contrast, free BDPE content varied markedly, with over 1000‐fold differences observed between products, indicating substantial variability in purification efficiency across manufacturing processes. Considerable variability was also identified among Food and Drug Administration–approved products, with some containing BDPE concentrations exceeding 100 ppm. Conversely, several products exhibited low BDPE levels ranging from 0.1 to 2.5 ppm, further highlighting inconsistencies in manufacturing control.

**Conclusions:**

Industry claims regarding complete cross‐linker removal may fail to account for the persistence of BDPE species. The substantial inter‐product variability observed in this study suggests inadequate process control among manufacturers. Given the structural similarity of BDPE to known sensitizers and the direct dermal injection route that circumvents the skin barrier, free BDPE should be designated a critical quality attribute with defined acceptance limits. These findings suggest that BDPE can be reduced to concentrations below 2.5 ppm, supporting the need for stricter manufacturing standards.

## 1 Introduction

**FIGURE 1:**
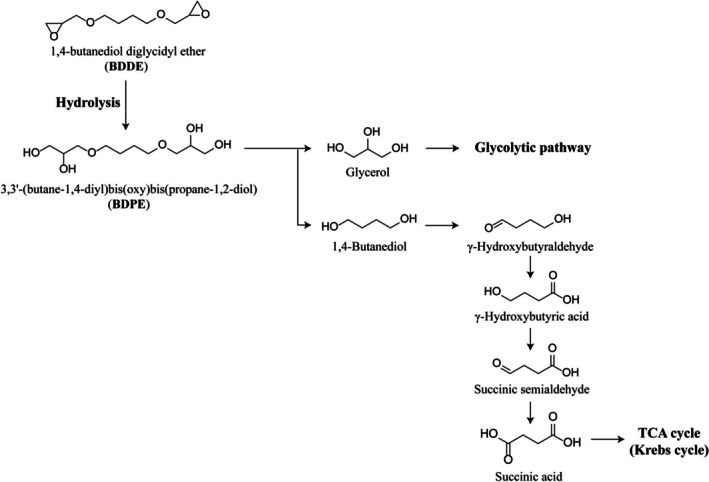
Metabolism of 1,4‐butanediol diglycidyl ether and its subsequent degradation products.

Hyaluronic acid (HA) dermal fillers constitute the most widely used category of minimally invasive aesthetic procedures worldwide, driven by increasing demand for non‐surgical facial rejuvenation. Their widespread clinical adoption has been attributed to favorable biocompatibility, reversibility with hyaluronidase, and predictable aesthetic outcomes [1, 2, 3]. Unlike permanent synthetic fillers, which are associated with risks such as granuloma formation and other long‐term complications, HA‐based products provide temporary tissue augmentation with well‐established safety profiles and are widely employed in clinical practice [4]. However, the inherently short in vivo half‐life of native HA—owing to rapid degradation by endogenous hyaluronidases—limits its clinical durability and necessitates chemical modification to achieve prolonged tissue persistence [5]. To mitigate enzymatic degradation, manufacturers commonly employ the chemical cross‐linker 1,4‐butanediol diglycidyl ether (BDDE) to generate HA networks with enhanced resistance to hyaluronidase activity [6, 7]. Following cross‐linking, residual BDDE undergoes hydrolysis at both epoxide groups under physiological and basic aqueous conditions, yielding 3,3′‐(butane‐1,4‐diyl)bis(oxy)bis(propane‐1,2‐diol) (BDPE; Figure 1).

Although unreacted BDDE is typically reduced to trace levels—generally below 2 ppm, the limit specified in the Food and Drug Administration (FDA) regulatory guidance—the presence, fate, and quantity of the hydrolyzed product BDPE have received limited systematic investigation [8, 9].

The 1,4‐butanediol moiety released during BDPE metabolism is sequentially oxidized by alcohol dehydrogenase to γ‐hydroxybutyraldehyde and subsequently by aldehyde dehydrogenase to γ‐hydroxybutyric acid. γ‐Hydroxybutyric acid is further metabolized by hydroxybutyric acid dehydrogenase and succinic semialdehyde dehydrogenase to produce succinic acid, which enters the Krebs cycle for cellular energy production (Figure 1). Glycerol, generated as a secondary metabolite, is metabolized through the glycolytic pathway [10].

The structural features of BDPE raise important safety considerations that have been inadequately addressed in the dermal filler literature. BDPE contains propane‐1,2‐diol substructures that are structurally analogous to propylene glycol and other polyol compounds known to cause concentration‐dependent skin irritation and contact dermatitis [11, 12, 13]. Propylene glycol, the compound most structurally similar to diol moieties of BDPE, has been associated with both allergic and irritant reactions in patch testing in a dose‐dependent manner and was designated the American Contact Dermatitis Society's Allergen of the Year in 2018 [14, 15]. In contrast to these well‐characterized polyols, which benefit from extensive safety data derived from decades of pharmaceuticals and cosmetic use, BDPE lacks comprehensive toxicological evaluation, despite its inevitable presence as a hydrolysis byproduct in BDDE‐cross‐linked HA fillers [9, 10, 16]. This deficiency in safety data is of particular concern given the unique exposure scenario of dermal fillers, in which direct intradermal or subcutaneous injection bypasses the stratum corneum barrier that normally limits systemic and local exposure to topically applied compounds.

In this study, we aimed to address the critical knowledge gaps concerning free BDPE content in commercial HA dermal fillers through a comprehensive analytical and safety assessment. A sensitive and validated liquid chromatography–tandem mass spectrometry (LC–MS/MS) method was developed and specifically optimized for BDPE quantification in HA filler matrices. Using this approach, products from major manufacturers representing the majority of the global filler market were systematically analyzed. Our findings provide the first comprehensive characterization of free BDPE content across commercial formulations and offer insights for optimizing filler safety through enhanced quality control measures.

## 2 Materials and Methods

### 2.1 Materials

All materials were commercially available and of analytical grade or higher. Detailed information is provided in the Supporting Information.

### 2.2 Chemical Toxicity Prediction

Skin irritation and skin sensitization potentials of BDPE, BDDE, 1,4‐butanediol, and glycerol were predicted using the OECD QSAR Toolbox (Version 4.7.1) and the VEGA QSAR platform (Version 1.2.4). For all compounds, SMILES (Simplified Molecular Input Line Entry System) codes were used as the standardized input format. The OECD QSAR Toolbox is a freely available software developed to facilitate reproducible and transparent chemical hazard assessment [17, 18]. It enables the prediction of toxicological properties through chemical grouping, category formation, and read‐across approaches based on mechanistic information and chemical similarity. In this study, the data gathering function was first used to compile available experimental and predicted toxicity information for each compound. Subsequently, the read‐across method was employed to evaluate both skin irritation and sensitization endpoints. The VEGA QSAR platform is an open‐source computational tool that incorporates more than 100 validated QSAR models [19]. It provides predictions for toxicological, ecotoxicological, environmental, and physicochemical endpoints, with reliability indicators to evaluate the confidence of each prediction. For skin irritation, predictions were obtained using the CONCERT/Coral model (v1.0.0). For skin sensitization, two models were applied: the CAESAR model (v2.1.7) and the NCSTOX model (v1.0.1). Each prediction generated by VEGA QSAR includes a reliability score, and in this study, only results specified as “good reliability” or based on experimental values were used.

Skin irritation and sensitization outcomes predicted by each software were compared and summarized alongside available experimental literature data for validation purposes.

### 2.3 Rheological Analysis

To evaluate the rheological properties of the HA filler samples, approximately 1 g of each sample was analyzed using a rheometer (Discovery HR20, TA Instruments, USA) equipped with a 40.0 mm, 2.0° cone‐plate geometry. The storage modulus (G') and phase angle were measured at 25°C across a frequency range of 0.01–100 Hz at 3.0% strain within the linear viscoelastic region, with values reported at 0.1 Hz.

### 2.4 Structural Confirmation of BDPE Standard

The identity and purity of the BDPE analytical standard were confirmed by ^1^H‐NMR spectroscopy and the manufacturer's certificate of analysis prior to use for LC–MS/MS quantification. For NMR analysis, 10 mg of the BDPE standard was dissolved in 1.0 mL of DMSO‐d_2_. ^1^H‐NMR spectroscopy was performed on a 400 MHz JEOL spectrometer (JNM‐ECZ400S/L1, Japan). The NMR spectra were processed and analyzed using MestReNova software (Mnova, Mestrelab Research, Spain).

### 2.5 LC–MS/MS Analysis of BDDE and BDPE

#### 2.5.1 Sample Preparation

Each sample (1.00 g) was enzymatically digested with hyaluronidase (580 U/mL, 0.5 mL) at 37°C for 12 h with shaking (300 rpm). The enzyme was inactivated at 95°C for 5 min. After dilution to 5 mL with purified water and centrifugation (4000 rpm, 20 min), the supernatant was filtered (0.22 μm) and further diluted (1–1000 fold) for LC–MS/MS analysis.

#### 2.5.2 LC–MS/MS Conditions

BDDE and BDPE were analyzed individually using an Agilent 1260 Infinity II LC system coupled to an Agilent 6470 LC/TQ mass spectrometer. Chromatographic separation was performed on an InfinityLab Poroshell 120 Aq‐C18 column (2.1 × 100 mm, 2.7 μm) at 40°C with a flow rate of 0.3 mL/min. Mobile phases consisted of (A) 0.1% formic acid and 2% acetonitrile in water and (B) 0.1% formic acid in acetonitrile. The gradient program was: 0–5 min, 100% A; 5–12 min, 100–60% A; 12–12.5 min, 60–0% A; 12.5–16 min, 0% A; 16–16.5 min, 0–100% A; 16.5–30 min, 100% A. The injection volume was 1 μL. Mass spectrometric detection was performed in positive electrospray ionization (ESI+) mode using multiple reaction monitoring (MRM). The MRM transitions were m/z 203.1 → 73.1 (quantifier) and 203.1 → 129.1 (qualifier) for BDDE, and m/z 239.0 → 73.0 (quantifier) and 239.0 → 147.0 (qualifier) for BDPE.

### 2.6 Statistics

All measurements were performed in triplicate unless otherwise stated. Data are presented as mean ± standard deviation (SD). Pearson's correlation coefficient was used to evaluate the relationship between free BDPE content and storage modulus (G'). Data analysis and visualization were performed using GraphPad Prism version 10.4 (GraphPad Software, San Diego, CA, USA) and Microsoft Excel (Microsoft Corporation, Redmond, WA, USA).

## 3 Results

### 3.1 Structural Confirmation of BDPE Standard

The spectrum confirms the molecular structure of BDPE (3,3′‐(butane‐1,4‐diyl)bis(oxy)bis(propane‐1,2‐diol)) with chemical formula C_10_H_22_O_6_ and molecular weight 238.28 g/mol (Figure S1). Proton assignments are detailed in the accompanying table showing chemical shifts, multiplicities, number of protons, and structural assignments for five distinct proton environments: δ 1.51–1.54 (m, 4H), δ 3.23–3.38 (m, 12H), δ 3.52–3.59 (m, 2H), δ 4.44–4.46 (t, 2H), and δ 4.57–4.58 (d, 2H) totaling 22 protons that confirm the expected structure (Table S1). The assignments are based on chemical shifts and multiplicities. The manufacturer's certificate of analysis (COA, Simson Pharma, India) confirmed BDPE standard purity of > 98% and molecular identity through mass spectrometry, showing a protonated molecular ion at m/z 239.2 [M + H]^+^ consistent with the theoretical molecular weight of 238.28 Da for C_10_H_22_O_6_.

### 3.2 Analytical Performance of the LC–MS/MS Method

The method was validated following ICH Q2(R2) guidelines. Calibration curves were linear (*R* ^2^ > 0.999) from 50–2000 ppb for both BDDE (y = 140.35x + 560.95) and BDPE (y = 198.05x + 145.91). Matrix effects were minimal with no interfering peaks detected. The limit of detection (LOD) and limit of quantification (LOQ) were determined by statistical evaluation using LINEST function analysis. For BDDE: LOD = 14.39 ppb, LOQ = 43.60 ppb (instrument level); considering dilution factor of 5 during sample preparation, actual sample LOD = 71.95 ppb, LOQ = 218.00 ppb. For BDPE: LOD = 15.13 ppb, LOQ = 45.85 ppb (instrument level); actual sample LOD = 75.65 ppb, LOQ = 229.25 ppb with dilution factor correction. Method precision was evaluated through multiple criteria: system precision %RSD ≤ 1%, intra‐laboratory precision (day‐to‐day variability) %RSD ≤ 10%, and inter‐analyst precision (analyst‐to‐analyst variability) %RSD ≤ 10% (Table S2).

### 3.3 Chemical Toxicity Prediction

Computational toxicity assessments for BDPE, BDDE, and their metabolites (1,4‐butanediol and glycerol) were performed using the OECD QSAR Toolbox and VEGA QSAR platform (Table 1). For skin irritation, both platforms predicted all four compounds as non‐irritating. The OECD QSAR Toolbox classified BDDE as not classified for irritation, while BDPE, 1,4‐butanediol, and glycerol were predicted as non‐irritating. Similarly, the VEGA QSAR model yielded non‐irritating predictions for all compounds. For skin sensitization, BDDE was predicted as a sensitizer by both OECD QSAR Toolbox and VEGA. Notably, BDPE was also predicted as positive for skin sensitization by OECD QSAR Toolbox and classified as a sensitizer by both VEGA QSAR models. In contrast, 1,4‐butanediol and glycerol were consistently predicted as non‐sensitizers across all models.

**TABLE 1:** Toxicological profiles and safety assessments of BDDE‐derived compounds and metabolites.

Material		BDPE	BDDE	1,4‐Butanediol	Glycerol
**SMILES**		C(CCOCC(CO)O) COCC(CO)O	C1C(O1)COCCCC OCC2CO2	C(CCO)CO	C(C(CO)O)O
**Structure**		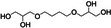	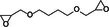	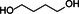	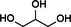
**Cytotoxicity**	Literature	Mouse fibroblast 300 ppm (80%) 480 ppm (50%) [9]	HaCaT – 100 ppm (40%) [20] HDF—100 ppm (70%) [20] HGF—20 ppm (49%) [21]	PC12 cell—450 ppm (83%) [22]	Non‐cytotoxic [23]
**Skin irritation**	Literature	**Skin irritating (GHS category 2)** [24]	**Skin irritating (GHS category 2)** [25, 26]	**Slightly irritating** [27, 28]	Non‐irritating [29, 30]
	OECD Toolbox	Not irritating	Not classified	Not irritating	Not irritating
	VEGA QSAR	Non‐irritating	Non‐irritating	Non‐irritating	Non‐irritating
**Skin sensitization**	Literature	**No information**	**Skin sensitizing (GHS category 1)** [25, 26]	Not sensitizing [27, 28]	Non‐sensitizing [30]
	OECD Toolbox	**Positive**	**Skin sensitizing**	Not sensitizing	Negative
	VEGA QSAR	**Sensitizer**	**Sensitizer**	Non‐sensitizer	Non‐sensitizer

Abbreviations: BDDE, 1,4‐butanediol diglycidyl ether; BDPE, 3,3′‐(butane‐1,4‐diyl)bis(oxy)bis(propane‐1,2‐diol); SMILES, simplified molecular input line entry system; HDF, human dermal fibroblast; HGF, human gingival fibroblast; GHS, globally harmonized system; OECD, organization for economic co‐operation and development; VEGA, virtual models for property evaluation of chemicals within a global architecture; QSAR, quantitative structure–activity relationship.

### 3.4 Residual BDDE and Free BDPE in Commercial HA Dermal Fillers

Using the validated LC–MS/MS method, residual BDDE and free BDPE were quantified in 38 commercial hyaluronic acid dermal fillers from seven manufacturers (Table 2). Residual BDDE was not detected in any of the tested products. In contrast, free BDPE content varied substantially among products, spanning more than three orders of magnitude from not detected to 646 ppm. Products manufactured using MPC technology consistently exhibited the lowest BDPE levels (≤ 2.5 ppm), whereas CPM and IPN‐Like technology products showed the highest concentrations (164–646 ppm). Substantial variability was also observed within the same manufacturer: products using OBT technology contained 10.8–22.6 ppm, while those using NASHA technology ranged from 56.1–121.3 ppm.

**TABLE 2:** Residual BDDE and BDPE content in commercial hyaluronic acid dermal fillers. Values represent mean ± SD from triplicate measurements of independent syringes from multiple lots (*n* = 3).

	BDDE residue (ppm)		BDPE residue (ppm)	
	Average	SD	Average	SD
Company A				
MPC‐TC technology				
MPC‐1	N.D.	—	**1.2**	0.13
MPC‐2	N.D.	—	**2.5**	0.22
MPC‐3	N.D.	—	**< 0.23**	—
MPC‐4	N.D.	—	**< 0.23**	—
MPC‐BY	N.D.	—	**0.4**	0.07
MPC‐R technology				
MPC‐F	N.D.	—	**1.3**	0.01
MPC‐D	N.D.	—	**N.D**.	—
MPC‐S	N.D.	—	**1.0**	0.16
MPC‐V10	N.D.	—	**1.1**	0.01
Company B				
Vycross technology				
VYC‐1	N.D.	—	**10.3**	1.43
VYC‐2	N.D.	—	**16.4**	0.05
VYC‐3	N.D.	—	**33.8**	0.16
VYC‐4	N.D.	—	**41.9**	4.42
VYC‐5	N.D.	—	**129.6**	0.18
Hylacross technology				
HYC‐1	N.D.	—	**69.2**	0.17
HYC‐2	N.D.	—	**97.4**	0.31
Company C				
OBT technology				
OBT‐1	N.D.	—	**10.8**	0.27
OBT‐2	N.D.	—	**13.5**	0.14
OBT‐3	N.D.	—	**22.6**	0.20
OBT‐4	N.D.	—	**15.4**	0.13
NASHA technology				
NASHA‐1	N.D.	—	**121.3**	0.68
NASHA‐2	N.D.	—	**109.8**	0.58
NASHA‐3	N.D.	—	**56.1**	0.47
Company D				
CPM technology				
CPM‐1	N.D.	—	**561.5**	7.10
CPM‐2	N.D.	—	**164.0**	1.93
CPM‐3	N.D.	—	**217.9**	1.42
CPM‐4	N.D.	—	**255.0**	2.49
CPM‐5	N.D.	—	**645.5**	0.81
Company E				
RHA technology				
RHA‐A	N.D.	—	**164.1**	0.84
RHA‐B	N.D.	—	**208.4**	0.81
RHA‐C	N.D.	—	**206.7**	0.97
Company F				
SMART technology				
SMART‐1	N.D.	—	**79.1**	6.63
SMART‐2	N.D.	—	**96.3**	6.48
SMART‐3	N.D.	—	**158.1**	12.1
Company G				
IPN‐Like technology				
IPN‐1	N.D.	—	**288.9**	1.63
IPN‐2	N.D.	—	**324.1**	2.21
IPN‐3	N.D.	—	**453.2**	4.59
IPN‐4	N.D.	—	**462.1**	4.80

Abbreviations: BDDE, 1,4‐butanediol diglycidyl ether; BDPE, 3,3′‐(butane‐1,4‐diyl)bis(oxy)bis(propane‐1,2‐diol); SD, standard deviation; ppm, parts per million; N.D., not detected.

### 3.5 Relationship Between Free BDPE Content and Rheological Properties

**FIGURE 2:**
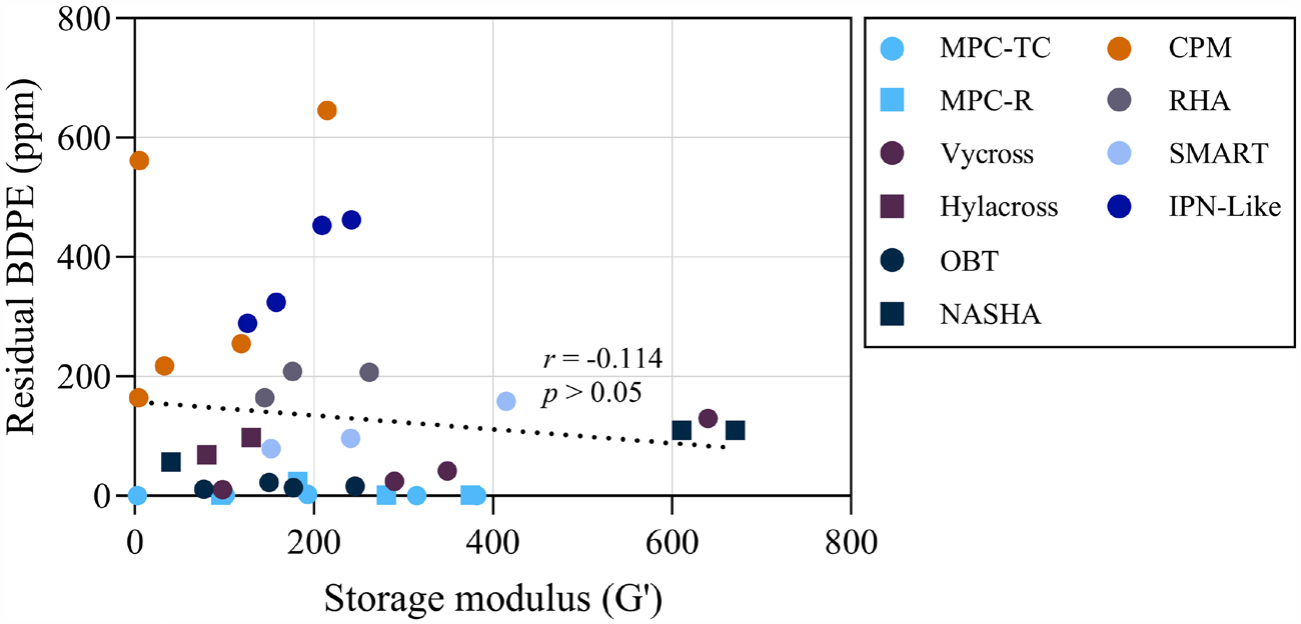
Correlation between storage modulus (G′, Pa) and residual BDPE content (ppm) in commercial hyaluronic acid fillers (38 products). The Pearson's correlation coefficient was *r* = −0.114 (*p* > 0.05), indicating no significant correlation.

To evaluate whether free BDPE content correlates with product characteristics, rheological analysis was performed across the same product panel (Table S3). The storage modulus (G′) ranged from 3 to 671 Pa and the phase angle from 5.5° to 53.9°, reflecting the diverse mechanical properties across product categories. Pearson's correlation analysis revealed no significant relationship between G′ and free BDPE content (*r* = −0.114, *p* > 0.05; Figure 2). Products with comparable G′ values exhibited markedly different BDPE concentrations, and conversely, products with similar BDPE content spanned a wide range of storage moduli.

## 4 Discussion

**FIGURE 3:**
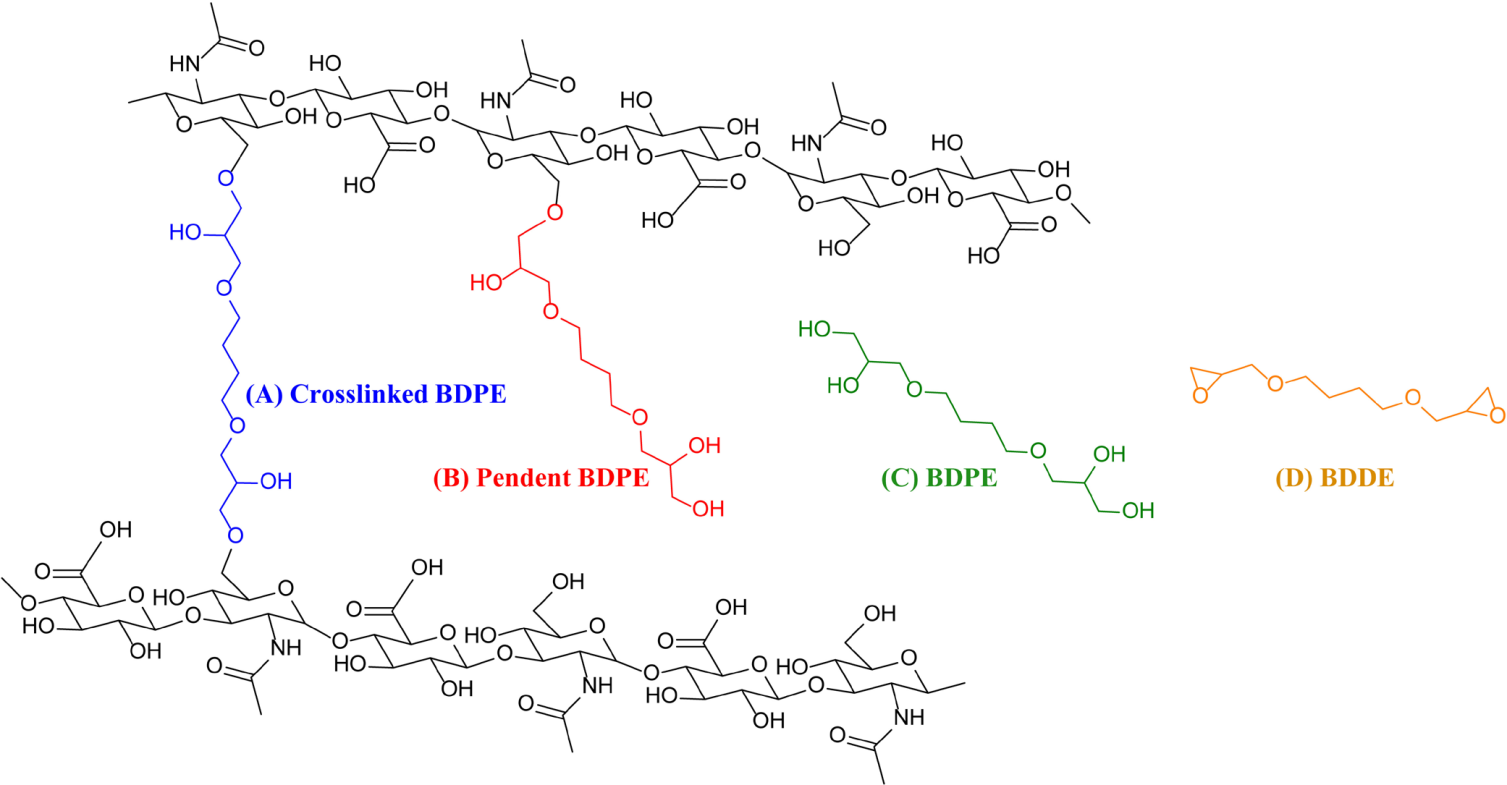
BDDE and its BDPE derivatives with various forms remained in hyaluronic acid‐based dermal fillers: (A) cross‐linked BDPE; (B) pendant BDPE; (C) BDPE, representing a hydrolyzed BDDE; and (D) residual BDDE.

Four distinct forms of BDPE derivatives as well as BDDE can be identified in the cross‐linked HA product: (A) cross‐linked BDPE, in which both terminal epoxide groups have reacted with separate HA chains to form inter‐chain cross‐links; (B) pendant BDPE, in which only one epoxide group has reacted with HA, leaving the other diol group as a side chain; (C) deactivated cross‐linker BDPE, representing a hydrolyzed BDDE that has not reacted with HA; and (D) residual BDDE (< 2 ppm), which remains in its unreacted native form (Figure 3). Risk assessment of BDDE‐derived residues must consider molecular weight, covalent attachment to the HA network, and consequent mobility and release potential. Among these compounds, free BDPE warrants particular attention: as a low‐molecular‐weight, unbound species, it exhibits high diffusivity within the gel matrix and readily elutes into surrounding tissue. By contrast, cross‐linked and pendant forms are covalently tethered to the polymer backbone and released only upon enzymatic degradation of the gel. The substantially higher concentrations of free BDPE relative to residual BDDE, combined with the absence of routine analytical monitoring for the hydrolyzed diol, highlight a previously underappreciated safety concern in BDDE‐crosslinked HA fillers.

Studies have reported free BDPE concentrations of 60–640 ppm [9] and 10–422 ppm [31] in a limited number of commercial products, exceeding the residual BDDE limit (< 2 ppm) by 5‐ to >300‐fold. Our analysis of 38 commercial products revealed comparable variability (Table 2). BDDE residues were non‐detectable across all tested products, confirming effective removal of unreacted crosslinker. By contrast, free BDPE content exhibited substantial variability among manufacturers, ranging from N.D. levels to 646 ppm. To evaluate whether BDPE content correlates with product characteristics, rheological analysis across the same product panel was performed. The storage modulus (G′), which reflects gel firmness and typically increases with greater cross‐linker input, was measured alongside other viscoelastic parameters (Table S3). Notably, G′ values showed no correlation with residual BDPE levels (*r* = −0.114, *p* > 0.05, Figure 2), indicating that BDPE content is primarily determined by purification efficiency rather than initial cross‐linker loading. These findings demonstrate that the effective removal of unreacted BDDE does not necessarily ensure low levels of its hydrolyzed byproduct, BDPE. Free BDPE content is predominantly determined by manufacturer‐specific purification efficiency rather than the degree of cross‐linking. Products utilizing advanced purification protocols, such as multi purified crosslinking (MPC) technology, exhibited substantially lower free BDPE levels (< 2.5 ppm), whereas several established brands displayed values ranging from 10 to over 600 ppm. This variation demonstrates the feasibility and necessity of enhanced post‐cross‐linking purification to improve the safety profile of HA dermal fillers.

Table 1 presents the currently available toxicological data for BDDE, BDPE. Both BDPE and BDDE demonstrated cytotoxicity in fibroblast cell lines at concentrations of 100–480 ppm, while glycerol showed no cytotoxicity [9, 20, 21, 22, 23]. BDDE is classified as an irritant (GHS category 2) and sensitizer (GHS category 1) based on experimental evidence, corroborated by computational predictions. BDPE demonstrates irritation potential per safety data sheet (GHS category 2); however, sensitization assessments rely exclusively on computational predictions (OECD Toolbox: Positive; VEGA QSAR: Sensitizer) due to the absence of experimental studies. This lack of experimental toxicological data for BDPE is particularly concerning given the concentrations observed in commercial fillers. Current safety assurances implicitly assume that hydrolysis of the epoxide groups substantially reduces biological reactivity—an assumption that remains unsupported by experimental evidence.

Although BDDE‐cross‐linked HA fillers have demonstrated an excellent overall safety record over two decades, persistent late‐onset adverse reactions continue to be reported [10, 32]. These events are frequently refractory to hyaluronidase treatment and cannot be attributed to contamination or the immunogenicity of HA itself, suggesting the involvement of manufacturing byproducts as potential contributing factors. Furthermore, a critical distinction lies in the route of administration: topical cosmetics are constrained by the stratum corneum barrier, whereas dermal fillers deliver BDPE directly into the dermis and subcutis, bypassing the skin's natural protective barrier. Given these concerns, a precautionary approach to free BDPE is warranted until robust safety data become available. Minimizing free BDPE through improved purification represents the most rational risk mitigation strategy.

**FIGURE 4:**
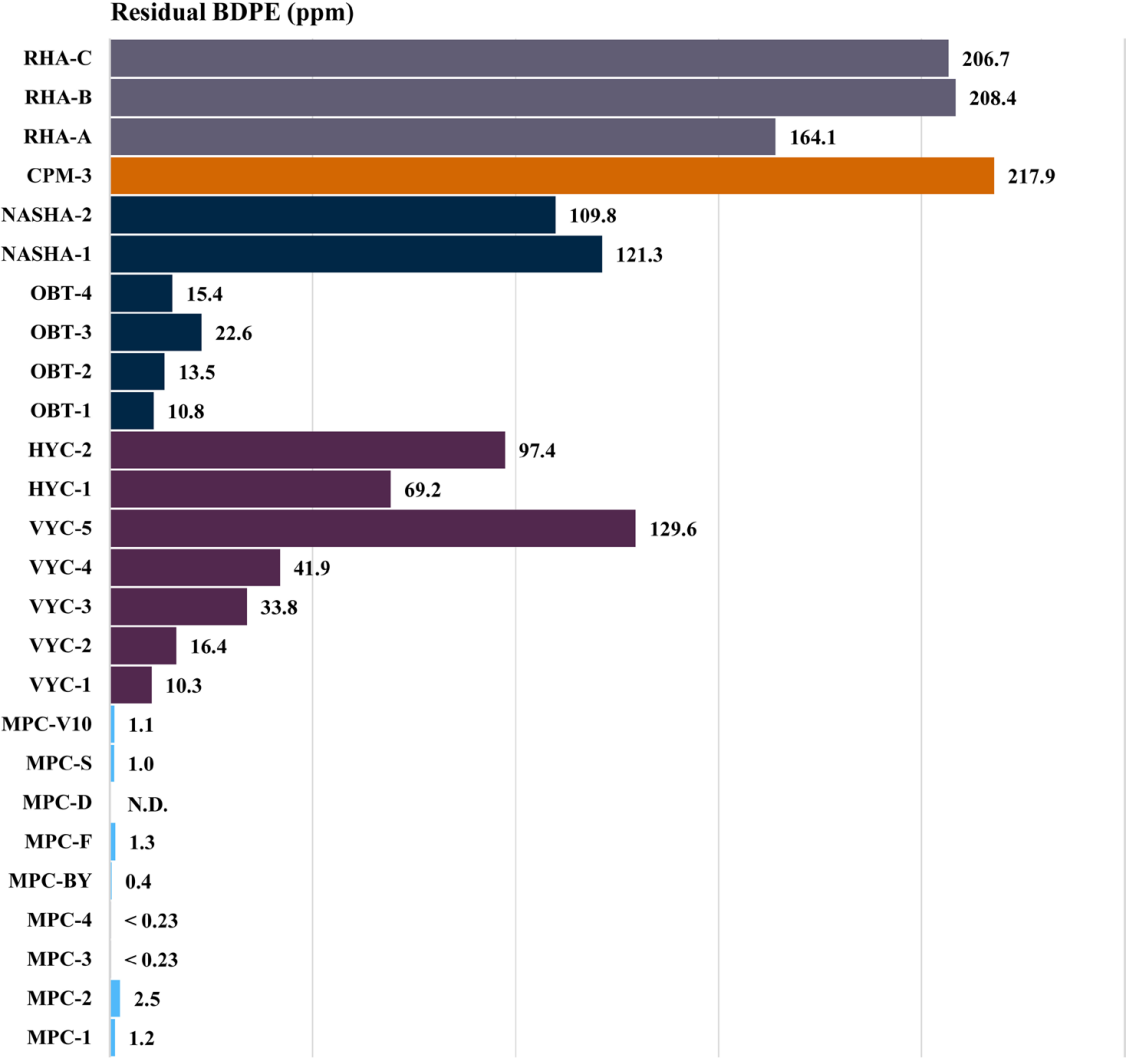
Comparison of free BDPE content across commercial dermal fillers: MPC‐processed versus FDA‐approved products.

Figure 4 presents a comparative analysis of free BDPE content across representative commercial products, including FDA‐approved dermal fillers and MPC‐processed formulations (selected from Table 2). MPC‐processed formulations demonstrated consistently low BDPE levels (N.D.–2.5 ppm), whereas major FDA‐approved products showed substantial variability, ranging from approximately 10 to over 200 ppm. This comparison demonstrates that, although all products comply with regulatory standards for unreacted BDDE removal, significant variability exists in the content of hydrolyzed byproducts—even among FDA‐approved formulations—reflecting differences in purification stringency among manufacturers. These findings indicate that near‐complete elimination of free BDPE is technically feasible through optimized post‐reaction purification. Collectively, free BDPE content should be considered a critical quality attribute with defined acceptance criteria, potentially informing future regulatory guidance for HA dermal filler manufacturing.

### 4.1 Study Limitations

This study has several limitations that should be acknowledged. First, although free BDPE content was comprehensively characterized and structural similarities to known sensitizers were identified, direct experimental evidence linking BDPE to clinical irritation remains limited. Available toxicological data for BDPE are largely restricted to in vitro cytotoxicity assays and computational predictions, with no controlled in vivo sensitization studies (e.g., direct peptide reactivity assay and human cell line activation test) conducted at relevant concentrations. Second, measurements were performed at a single time point on marketed products and do not account for potential changes during long‐term storage or in vivo release kinetics of BDPE following injection. Third, although substantial inter‐manufacturer variability was observed, the proprietary nature of manufacturing processes precluded the identification of the specific purification parameters responsible. Finally, long‐term clinical outcomes comparing high‐BDPE versus low‐BDPE formulations were not evaluated.

## 5 Conclusion

This study demonstrates that current regulatory and industry focus on residual BDDE alone (< 2 ppm) is insufficient to ensure complete removal of cross‐linker byproducts. Substantial quantities of the hydrolyzed derivative, free BDPE, persist in commercial HA dermal fillers at concentrations spanning more than three orders of magnitude, with many leading products exceeding 100 ppm despite full compliance with existing BDDE limits. The pronounced variability in free BDPE content—exceeding 1000‐fold between products—indicates that current manufacturing processes achieve markedly different purification outcomes. Despite the limitations of this work—notably the lack of direct clinical correlation and comprehensive in vivo toxicological data—the results demonstrate that free BDPE concentrations below 2.5 ppm are technically achievable using current manufacturing technology without compromising product performance. The availability of such low‐BDPE formulations confirms that substantial byproduct reduction is feasible.

Therefore, free BDPE may warrant consideration as a regulated critical quality attribute for BDDE‐cross‐linked HA fillers, with dedicated analytical monitoring and defined acceptance criteria incorporated into future regulatory guidelines. Implementation of enhanced post‐cross‐linking purification protocols capable of routinely reducing free BDPE to negligible levels will further strengthen the long‐term safety profile of these widely used aesthetic products.

## Author Contributions

Cheol Joo Kim conceived and designed the study, performed data analysis, and drafted the manuscript. Seung Jun Shin and Cheolho Shin contributed to the conception and design of the study, supervised the research, and served as co‐corresponding authors. Mirou Lee contributed substantially to data analysis and interpretation and critically revised the manuscript. Hyeongtaek Park, Jaeyoung Jo, Hyolim Lee, and Pius J. Seo contributed to data acquisition, analysis, and interpretation and provided critical scientific input. All authors critically revised the manuscript for important intellectual content, approved the final version, and agree to be accountable for all aspects of the work.

## Ethics Statement

The authors have nothing to report.

## Conflicts of Interest

The authors declare no conflicts of interest.

## Supporting information


**Data S1:** supporting Information.

## Data Availability

The data that support the findings of this study are available from the corresponding author upon reasonable request.
