# Supplementary material for: Comprehensive Analysis of Free BDPE Content in Commercial Hyaluronic Acid Fillers: Implications for Safety Assessment and Regulatory Standards
Source: J Cosmet Dermatol. 2026 Mar 10;25(3):e70790. doi: 10.1111/jocd.70790 (PMC12973259; doi:10.1111/jocd.70790)
Supplement: Supplementary file 1 — Data S1: supporting Information. [file JOCD-25-e70790-s001.docx]

*Supporting Information*

*for*

Comprehensive Analysis of Free BDPE Content in

Commercial Hyaluronic Acid Fillers

: Implications for Safety Assessment and Regulatory Standards

**Materials**

1,4-Butanediol diglycidyl ether (BDDE, catalog no. 220892) was purchased from Sigma-Aldrich (USA) and used as an analytical standard. 3,3'-(Butane-1,4-diyl)bis(oxy)bis(propane-1,2-diol) (BDPE, catalog no. G340017) was purchased from Simson Pharma (India) and used as an analytical standard for LC-MS/MS quantification. Hydrochloric acid (HCl, catalog no. 4090-4400) was purchased from DAEJUNG (South Korea). Sodium hydroxide (NaOH, catalog no. 1636) was purchased from DUKSAN (South Korea). HPLC-grade acetonitrile (ACN, catalog no. 9017-88), methanol (MeOH, catalog no. 9093-88), and distilled water (catalog no. 4218-88) were purchased from Avantor (USA).

The commercial hyaluronic acid fillers used in this study were obtained from different suppliers as follows: Across (The Chaeum^®^ series (No.1, No.2, No.3, No.4), Revolax^®^ series (Fine, Deep, SubQ), BYRYZN^®^, and Volus^®^ 10, South Korea), Allergan (Juvéderm^®^ series (Skinvive, Ultra XC, Ultra Plus XC, Vobella, Volift, Voluma, Volux), USA), Galderma (Restylane^®^ series (Refyne, Kysse, Volyme, Defyne, Lidocaine, Lyft, Vital Light), Switzerland), Merz (Belotero^®^ series (Revive, Soft, Balance, Intense, Volume), Germany), Teoxane (Teosyal^®^ series (RHA 2, RHA 3, RHA 4), Switzerland), Croma (Saypha^®^ series (Filler-L, Volume-L, Volume Plus), Austria), Vivacy (Stylage^®^ series (S, M, L, XL), France). All products were stored according to manufacturer specifications and used before expiration dates.


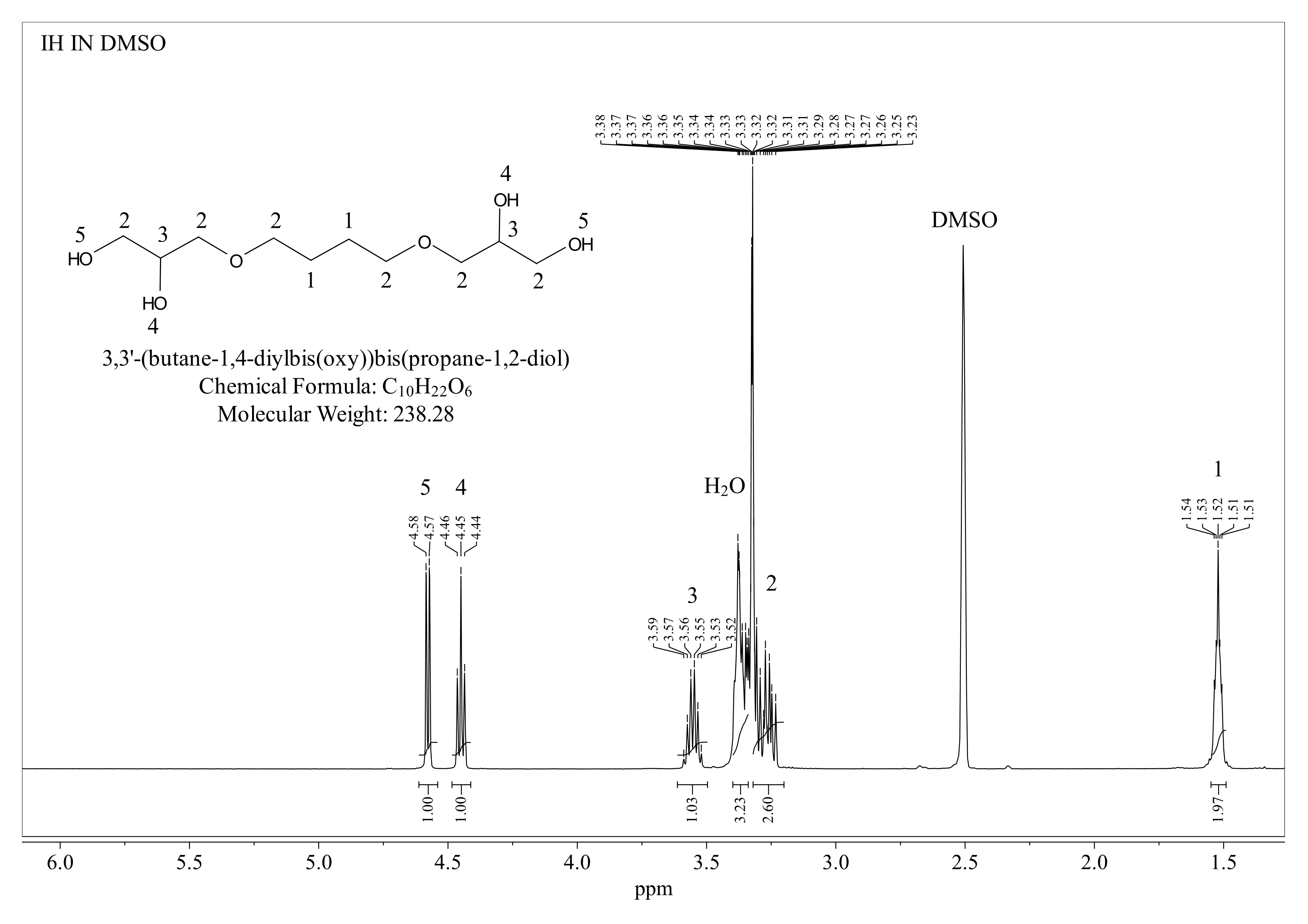


**Figure S1.** ¹H-NMR spectrum of BDPE standard in DMSO-d₆.

**Table S1.** ^1^H-NMR peak assignment of BDPE standard.

| **No.** | **Chemical shift δ, (ppm)** | **Multiplicity** | **Number of protons** | **Proton assignment** |  |
| --- | --- | --- | --- | --- | --- |
| 1 | 1.51 - 1.54 | M | 4H | 4 | |
| 2 | 3.23 - 3.38 | M | 12H | 12 | |
| 3 | 3.52 - 3.59 | M | 2H | 2 | |
| 4 | 4.44 - 4.46 | T | 2H | 2 | |
| 5 | 4.57 - 4.58 | D | 2H | 2 | |
| Total number of protons | | | | 22 | |

**Table S2.** Method validation parameters for LC-MS/MS quantification of BDDE and BDPE

| **Parameter** | | **BDDE** | **BDPE** |
| --- | --- | --- | --- |
| Recovery (%, n=9) | | 100.3 ± 2.5 | 100.3 ± 1.9 |
| LOD (**ppb**) | Instrument level | 14.39 | 15.13 |
|  | Actual sample level | 71.95 | 75.65 |
| LOQ (**ppb**) | Instrument level | 43.60 | 45.85 |
|  | Actual sample level | 218.00 | 229.23 |
| Precision (RSD, %) | System | < 1 | < 1 |
|  | Intra-laboratory  (day-to-day variability) | < 10 | < 10 |
|  | Inter-analyst precision  (analyst-to-analyst variability) | < 10 | < 10 |

**Table S3.** Rheological properties of commercial hyaluronic acid dermal fillers

|  | **HA conc. (mg/mL)** | **Rheological properties (at 0.1 Hz)** | | | | |
| --- | --- | --- | --- | --- | --- | --- |
|  |  | **G’ (Pa)** | | | **Phase angle (^o^)** | |
| **Company A** |  |  |  |  | |  |
| **MPC-TC technology** |  |  |  |  | |  |
| MPC-1 | **24** | 101 | | | 16.4 | |
| MPC-2 | **20** | 193 | | | 9.2 | |
| MPC-3 | **20** | 315 | | | 7.6 | |
| MPC-4 | **20** | 382 | | | 7.9 | |
| MPC-BY | **20** | 3 | | | 44.6 | |
| **MPC-R technology** |  |  | | |  | |
| MPC-F | **24** | 96 | | | 18.3 | |
| MPC-D | **24** | 182 | | | 13.7 | |
| MPC-S | **24** | 281 | | | 6.7 | |
| MPC-V10 | **20** | 375 | | | 7.3 | |
| **Company B** |  |  | | |  | |
| **Vycross technology** |  |  | | |  | |
| VYC-1 | **12** | 98 | | | 18.8 | |
| VYC-2 | **15** | 246 | | | 11.7 | |
| VYC-3 | **17.5** | 290 | | | 10.2 | |
| VYC-4 | **20** | 349 | | | 7.4 | |
| VYC-5 | **25** | 640 | | | 6.8 | |
| **Hylacross technology** |  |  | | |  | |
| HYC-1 | **24** | 80 | | | 12.1 | |
| HYC-2 | **24** | 130 | | | 10.1 | |
| **Company C** |  |  | | |  | |
| **OBT technology** |  |  | | |  | |
| OBT-1 | **20** | 77 | | | 10.4 | |
| OBT-2 | **20** | 177 | | | 6.3 | |
| OBT-3 | **20** | 150 | | | 5.7 | |
| OBT-4 | **20** | 246 | | | 5.5 | |
| **NASHA technology** |  |  | | |  | |
| NASHA-1 | **20** | 611 | | | 14.5 | |
| NASHA-2 | **20** | 671 | | | 11.3 | |
| NASHA-3 | **12** | 40 | | | 25.9 | |
| **Company D** |  |  | | |  | |
| **CPM technology** |  |  | | |  | |
| CPM-1 | **20** | 5 | | | 53.9 | |
| CPM-2 | **20** | 4 | | | 51.0 | |
| CPM-3 | **22.5** | 33 | | | 27.7 | |
| CPM-4 | **25.5** | 119 | | | 16.9 | |
| CPM-5 | **26** | 215 | | | 11.6 | |
| **Company E** |  |  | | |  | |
| **RHA technology** |  |  | | |  | |
| RHA-A | **23** | 145 | | | 14.0 | |
| RHA-B | **23** | 176 | | | 9.0 | |
| RHA-C | **23** | 262 | | | 7.7 | |
| **Company F** |  |  | | |  | |
| **SMART technology** |  |  | | |  | |
| SMART-1 | **23** | 152 | | | 8.8 | |
| SMART-2 | **23** | 241 | | | 5.7 | |
| SMART-3 | **25** | 415 | | | 5.2 | |
| **Company G** |  |  | | |  | |
| **IPN-Like technology** |  |  | | |  | |
| IPN-1 | **16** | 126 | | | 10.0 | |
| IPN-2 | **20** | 158 | | | 10.4 | |
| IPN-3 | **24** | 209 | | | 7.3 | |
| IPN-4 | **26** | 242 | | | 7.8 | |
